# Intratumoral microbiota drive immune evasion and disease progression in oral squamous cell carcinoma

**DOI:** 10.1038/s41405-025-00385-x

**Published:** 2025-12-01

**Authors:** Xiaolong Zang, Xiaoxia Li, Rongxin Sun, Xiaojie Zhang, Zhiyong Li, Zijian Cheng

**Affiliations:** Stomatology Hospital, School of Stomatology, Zhejiang University School of Medicine, Zhejiang Provincial Clinical Research Center for Oral Diseases, Zhejiang Key Laboratory of Oral Biomedical, Hangzhou, China

**Keywords:** Oral cancer, Oral cancer detection, Oral cancer detection

## Abstract

**Objective:**

Emerging evidence suggests that oral squamous cell carcinoma (OSCC) harbors distinct microbial communities, yet their influence on tumor immunobiology remains unclear. This study investigated the prognostic value of intratumoral microbiota and their role in modulating CD8⁺ T-cell function.

**Materials and methods:**

Using TCGA datasets, a 19-microorganism prognostic signature was constructed via Cox and LASSO regression and validated with Kaplan–Meier survival analysis. Comprehensive bioinformatics analyses, including GSEA/GSVA, CIBERSORT, ESTIMATE, pharmacogenomics, and mutational profiling, were performed to explore underlying mechanisms.

**Results:**

The microbial signature demonstrated strong predictive performance, with higher risk scores significantly associated with reduced overall survival (p < 0.001). High-risk tumors exhibited enrichment of PI3K/AKT/mTOR signaling, metabolic reprogramming, and epithelial–mesenchymal transition, alongside an immunosuppressive microenvironment characterized by CD8⁺ T-cell depletion, M0 macrophage infiltration, and upregulation of immunosuppressive markers including CD276 and TGF-β1. Conversely, immune-activating checkpoints such as PD-1 and CTLA-4 were elevated in low-risk tumors. Notably, periodontal pathogens negatively correlated with immune effector activity, and TP53 mutations were more frequent in high-risk cases (82% vs. 67%).

**Conclusions:**

This study identified intratumoral microbial signatures as independent prognostic biomarkers and validated their reproducibility in an external cohort. Our findings support a microbiota–immune axis contributing to immune evasion in OSCC, offering novel avenues for prognostic stratification and therapeutic intervention.

## Introduction

Oral squamous cell carcinoma (OSCC) is a major global health burden, with 389,346 new cases reported in 2022 according to Global Cancer Observatory statistics [1]. Despite advances in surgery, radiotherapy, and chemotherapy, the 5-year survival rate remains approximately 64%, particularly low among patients with advanced-stage disease [2–4]. Understanding the molecular and microenvironmental factors that drive OSCC progression remains essential to improving both prognosis and treatment outcomes.

Emerging evidence suggests that the intratumoral microbiota actively shapes tumor biology by influencing carcinogenesis, immune modulation, and therapy response [5]. The oral microbiome constitutes a highly diverse ecosystem, where complex host–microbe interactions influence both health and disease states [6]. Recent findings suggested that specific taxa, such as *Fusobacterium nucleatum* and *Prevotella intermedia*, could promote epithelial–mesenchymal transition, immune evasion, and tumor progression in OSCC, indicating that microbial signatures within the tumor microenvironment are not merely passive residents but potential drivers and biomarkers of disease development [7]. Oyeyemi et al. showed in an Indian cohort that the saliva of OSCC patients and tobacco abusers exhibited increased abundance of *Campylobacter* and *Leptotrichia*, highlighting potential non-invasive microbiome-based biomarkers for early oral cancer screening [8]. However, whether these microbial shifts are causal drivers or secondary byproducts of tumorigenesis remains controversial. In particular, the mechanisms underlying microbiota–immune crosstalk in OSCC are still poorly understood.

High-throughput sequencing and integrative bioinformatics now allow systematic exploration of tumor–microbe interactions, enabling deeper insights into how microbial communities contribute to oncogenic signaling and immune modulation [9]. The Cancer Genome Atlas (TCGA) provides a unique opportunity to integrate genomic and microbiome data to explore these relationships in OSCC [10].

In this study, we systematically analyzed TCGA datasets to investigate the prognostic significance of intratumoral microbiota in OSCC and to examine their potential influence on the immune microenvironment. We developed and validated a 19-microorganism prognostic signature that stratifies patients into high- and low-risk groups and explored associated molecular pathways and immune features. Our findings reveal a potential microbiota–immune axis in OSCC pathogenesis, providing new evidence that microbial signatures may serve as both prognostic biomarkers and therapeutic targets for personalized treatment.

## Materials And Methods

### Data acquisition

Transcriptomic and clinical data for oral squamous cell carcinoma (OSCC) were obtained from The Cancer Genome Atlas (TCGA) database (https://portal.gdc.cancer.gov/), which provides the largest publicly available repository of cancer genomic data, including gene expression, miRNA expression, copy number variations, DNA methylation, and SNP profiles. Processed mRNA expression data comprising 340 tumor samples and 32 adjacent normal samples were downloaded for analysis.

### Intratumoral microbiome profiling

Intratumoral microbial profiles were obtained from a previously published study using Kraken for taxonomic classification [11, 12]. Kraken is a k-mer–based metagenomic sequence classifier with high speed and accuracy. Associations between microbial abundance and host features were further identified by integrative analyses linking microbiota with immune microenvironment composition, gene expression, and predicted immunotherapy response [13].

### Prognostic model construction

A total of 534 bacterial genera detected in OSCC samples were screened for prognostic relevance using univariate Cox proportional hazards regression [14]. Patients were randomly divided into a training set and an internal validation set at a 4:1 ratio. Least absolute shrinkage and selection operator (LASSO) regression was applied to construct a multivariate prognostic model, and a risk score for each patient was calculated as the weighted sum of microbial abundances multiplied by their regression coefficients. Patients were stratified into high- and low-risk groups based on the median risk score. Survival differences were assessed using Kaplan–Meier analysis with log-rank testing. Model performance was evaluated using time-dependent receiver operating characteristic (ROC) curves and validated in stratified subgroups.

### Immune cell infiltration analysis

The relative abundance of 22 immune cell subsets was estimated using the CIBERSORT algorithm, which applies support vector regression to deconvolute bulk RNA-seq data based on a signature matrix of 547 marker genes distinguishing T cells, B cells, plasma cells, macrophages, dendritic cells, and other leukocyte subsets [14].

### Drug sensitivity prediction

Chemotherapeutic sensitivity was inferred using the Genomics of Drug Sensitivity in Cancer (GDSC) database (https://www.cancerrxgene.org/) with the R package pRRophetic. Half-maximal inhibitory concentrations (IC50) for each drug were estimated via ridge regression, and prediction accuracy was assessed using 10-fold cross-validation against the GDSC training dataset.

### Gene set enrichment analyses

Gene set variation analysis (GSVA) was performed using gene sets from the Molecular Signatures Database (MSigDB v7.0) to score pathway activity across samples [15]. Differential pathway enrichment between risk groups was further evaluated by gene set enrichment analysis (GSEA; http://www.broadinstitute.org/gsea) with 1000 permutations. Gene sets with 15–500 members were considered, and enrichment was deemed significant at *P* < 0.05 and false discovery rate (FDR) < 0.25.

### Statistical analysis

All statistical analyses were conducted in R (v4.0). Survival outcomes were analyzed using Kaplan–Meier curves with log-rank tests. Unless otherwise specified, all *P*-values were two-sided, and *P* < 0.05 was considered statistically significant.

## Results

### Intratumoral bacterial microbiome in OSCC

The intratumoral bacterial composition of OSCC and adjacent normal tissues was profiled to assess microbial alterations. At the phylum, class, and genus levels, the top 10 taxa were comparable between groups, with no significant differences observed (Fig. A1A–C). However, differential abundance analysis identified 534 genera significantly enriched or depleted in OSCC tumors (*P* < 0.05), indicating a markedly altered microbial landscape. Nine representative genera with the most significant changes are shown in Fig. A1D. Despite these compositional shifts, alpha diversity indices (Shannon, Simpson, and Pielou’s evenness) revealed no significant differences between tumor and control samples (Fig. A1E).

### Identification of Prognostic Microorganisms and Construction of a Predictive Model

**Fig. 1:**
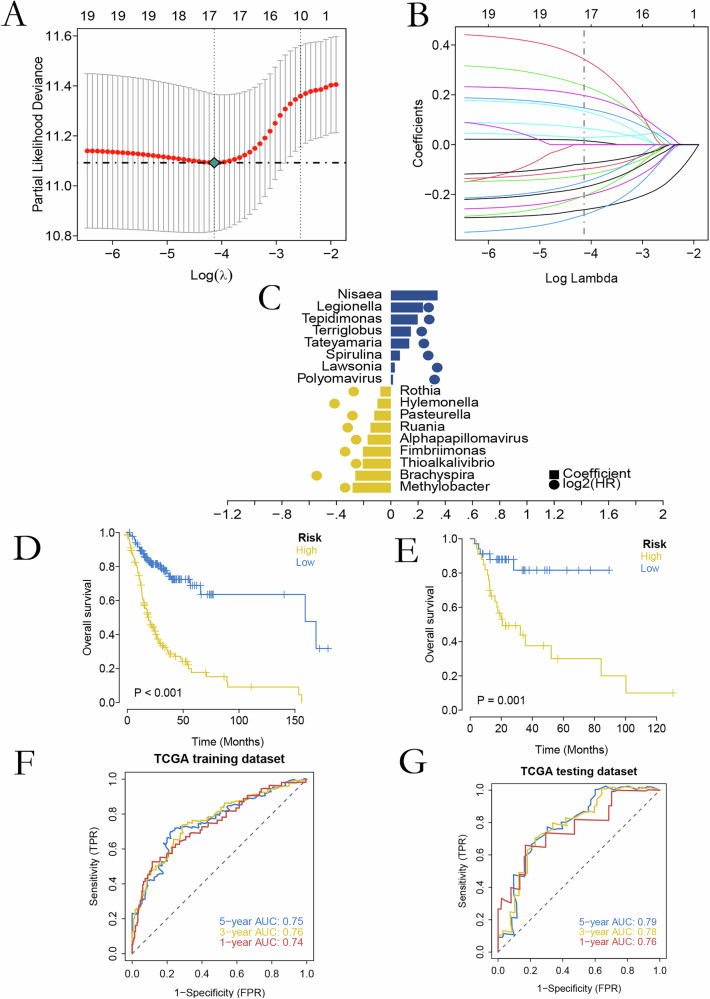
Development of an OSCC prognostic model based on intratumoral microbiota. **A** LASSO model λ selection via fivefold cross-validation. Dotted lines indicate minimum and 1-SE criteria. **B** Coefficient profiles of 19 bacteria showing shrinkage toward zero with increasing penalty. **C** We obtained the best risk score value (17 out of 19 prognosis-related microorganisms) corresponding to each sample for subsequent correlation analysis. **D**, **E** Kaplan–Meier curves comparing OS between high- and low-risk groups in training/testing cohorts. **F**, **G** Time-dependent ROC curves with 1-, 3-, and 5-year AUCs for training/testing sets.

Univariate Cox regression identified 19 microorganisms significantly associated with overall survival (*P* < 0.05). These taxa were integrated into a LASSO-derived risk model, generating a microbial risk score for each patient (Fig. 1A–C). Based on the median risk score, patients were stratified into high- and low-risk groups. Kaplan–Meier analysis demonstrated significantly worse survival in the high-risk group in both training and validation cohorts (Fig. 1D–E). Time-dependent ROC curves yielded AUCs ≥ 0.74 at 1, 3, and 5 years in both sets, confirming strong predictive performance (Fig. 1F–G).

### Model validation and clinical correlation

**Fig. 2:**
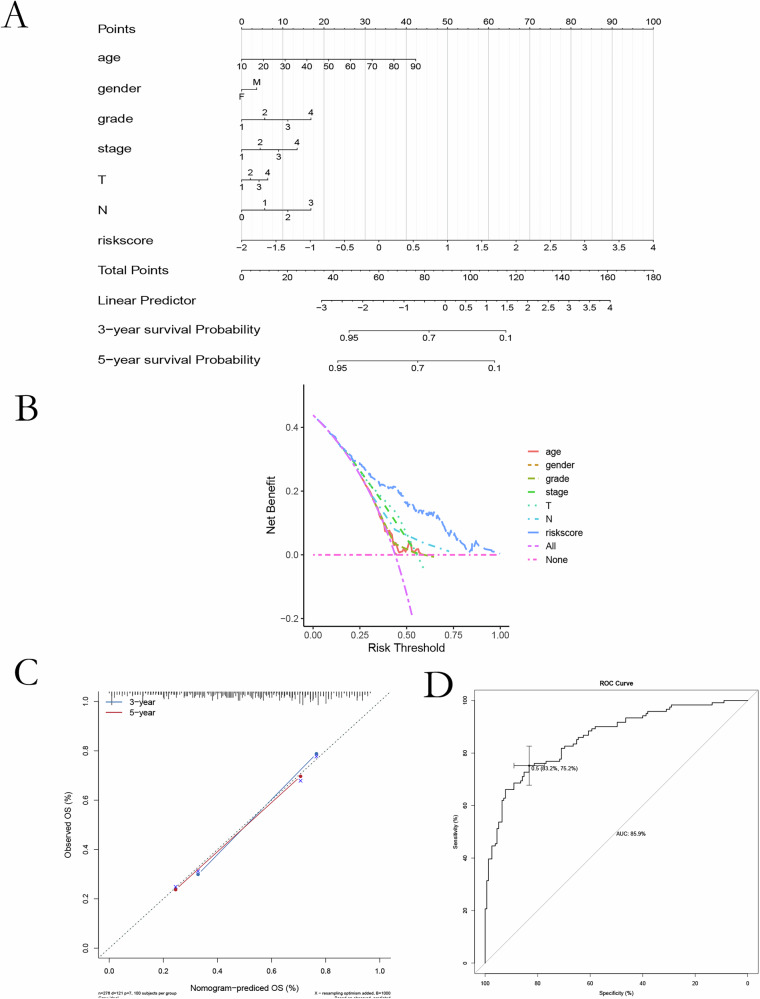
Risk scoring and prognostic evaluation in OSCC. **A** Nomogram combining clinical factors (age, grade, T/N stage) and microbial risk scores for OS prediction. **B** Decision-curve analysis (DCA) showing net benefit of the combined model. **C** Calibration curves for 3- and 5-year survival agreement between predicted and observed outcomes. **D** ROC analysis validating high predictive accuracy of the risk-score model.

**Fig. 3:**
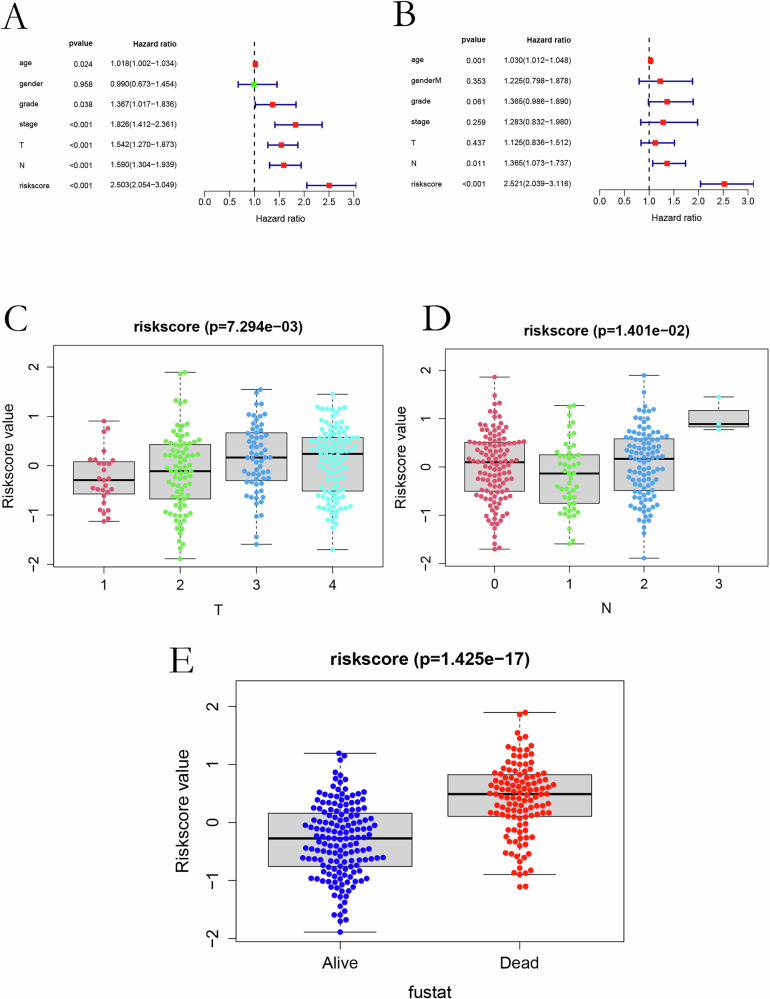
Cox regression and clinical correlates of the microbial risk score. **A**, **B** Forest plots of univariate and multivariate Cox analyses showing hazard ratios and 95% CIs for clinical variables and microbial risk score. **C**–**E** Associations between the risk score and tumor (T) stage, nodal (N) status, and survival outcome.

A nomogram integrating microbial risk scores with clinicopathological parameters (age, tumor grade, and nodal status) demonstrated robust prognostic value (Fig. 2A). Decision curve analysis confirmed the added clinical utility of the risk model compared to individual variables (Fig. 2B). Calibration curves for 3- and 5-year survival showed excellent agreement between predicted and observed outcomes (Fig. 2C). ROC analysis further validated the model with AUCs ≥ 0.75 (Fig. 2D). Univariate and multivariate Cox regression analyses identified the microbial risk score as an independent prognostic factor (*P* < 0.001) (Fig. 3A, B). Higher scores were significantly associated with advanced T stage, nodal involvement, distant metastasis, and poor survival status (Fig. 3C–E).

### Differential pathway enrichment between risk groups

**Fig. 4:**
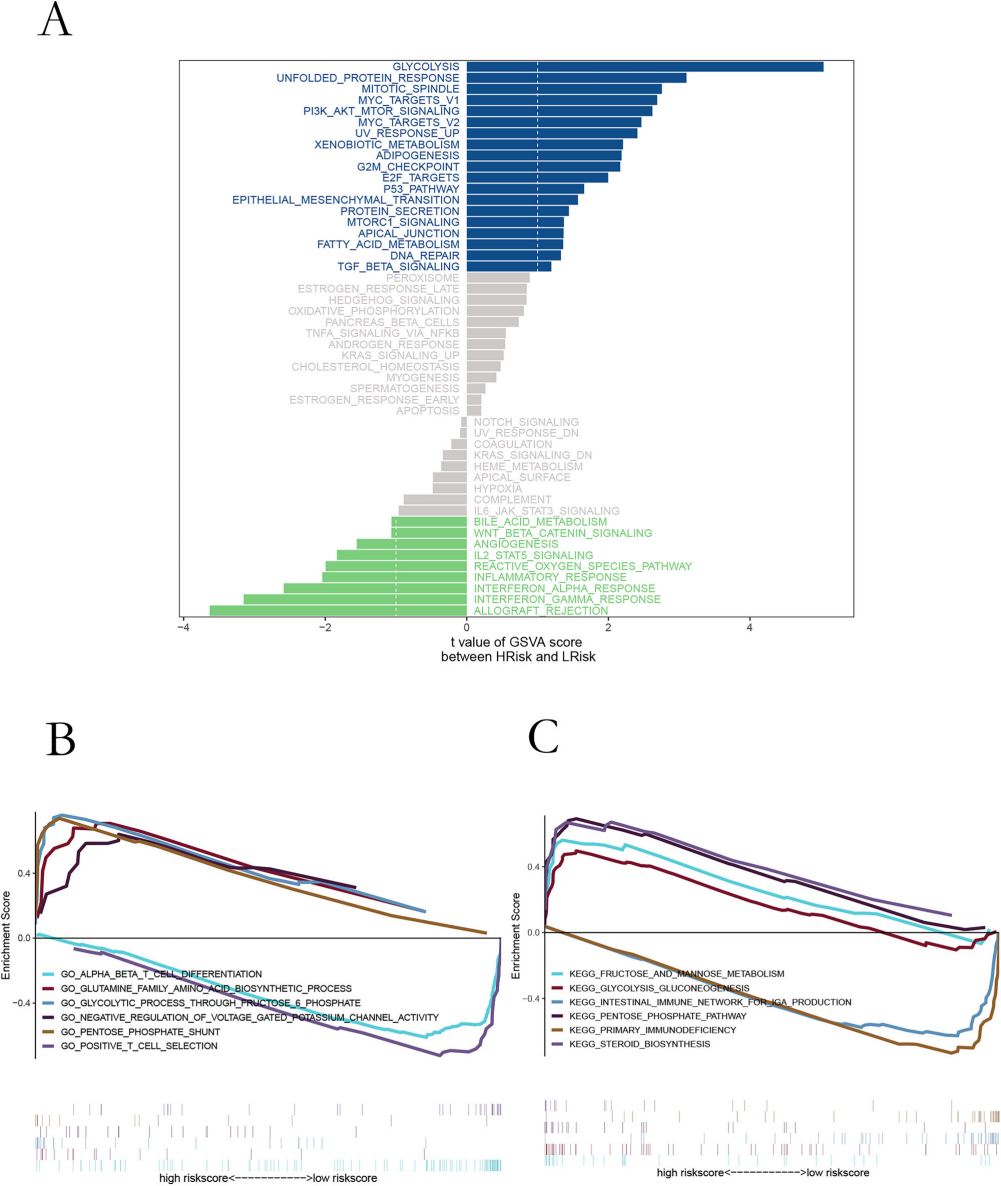
Pathway analyses associated with microbial risk. **A** GSVA comparison showing distinct pathway activation between risk groups. **B** GO enrichment highlighting biological processes linked to differential risk. **C** KEGG analysis identifying key signaling and metabolic pathways enriched in high- vs low-risk tumors.

**Fig. 5:**
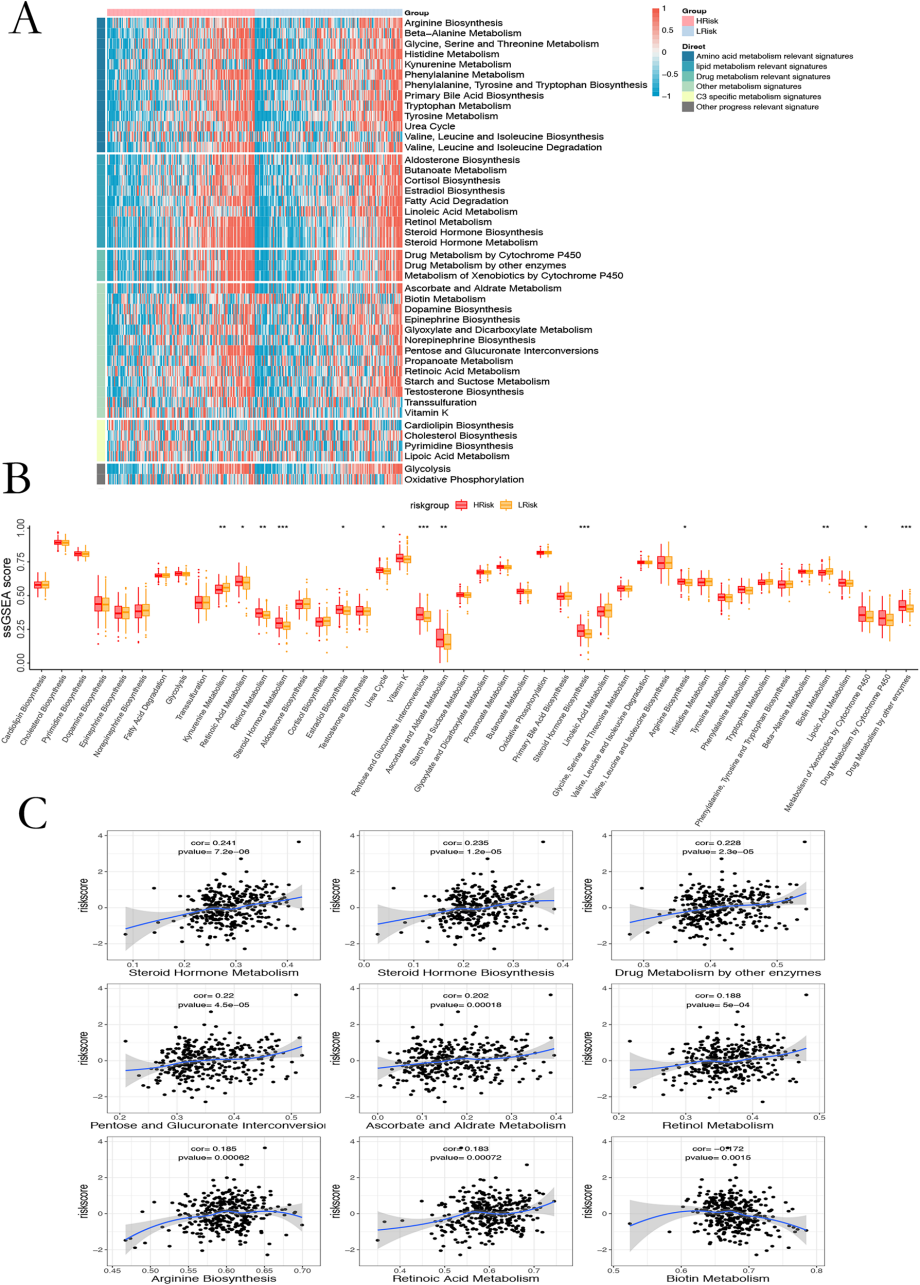
Metabolic pathway enrichment. **A** Heatmap of 44 metabolic pathways (blue–red gradient). **B** ssGSEA enrichment scores showing risk-associated metabolic alterations (*p* < 0.05–0.001). **C** Correlations between risk scores and enriched pathways with corresponding *p*-values.

GSVA revealed that glycolysis, PI3K/AKT/mTOR signaling, mitotic spindle assembly, and epithelial–mesenchymal transition were significantly enriched in the high-risk group (Fig. 4A). GSEA corroborated these findings and identified additional pathway differences, including enrichment of glutamine biosynthesis and pentose phosphate shunt (GO) and fructose/mannose metabolism, intestinal immune network for IgA production, and steroid biosynthesis (KEGG) in high-risk tumors (Fig. 4B, C). Metabolism-focused analyses showed retinoic acid metabolism, retinol metabolism, and steroid hormone pathways markedly upregulated in high-risk samples (Fig. 5A, B). Risk scores correlated positively with steroid hormone biosynthesis and negatively with biotin metabolism (Fig. 5C).

### Tumor microenvironment characteristics

**Fig. 6:**
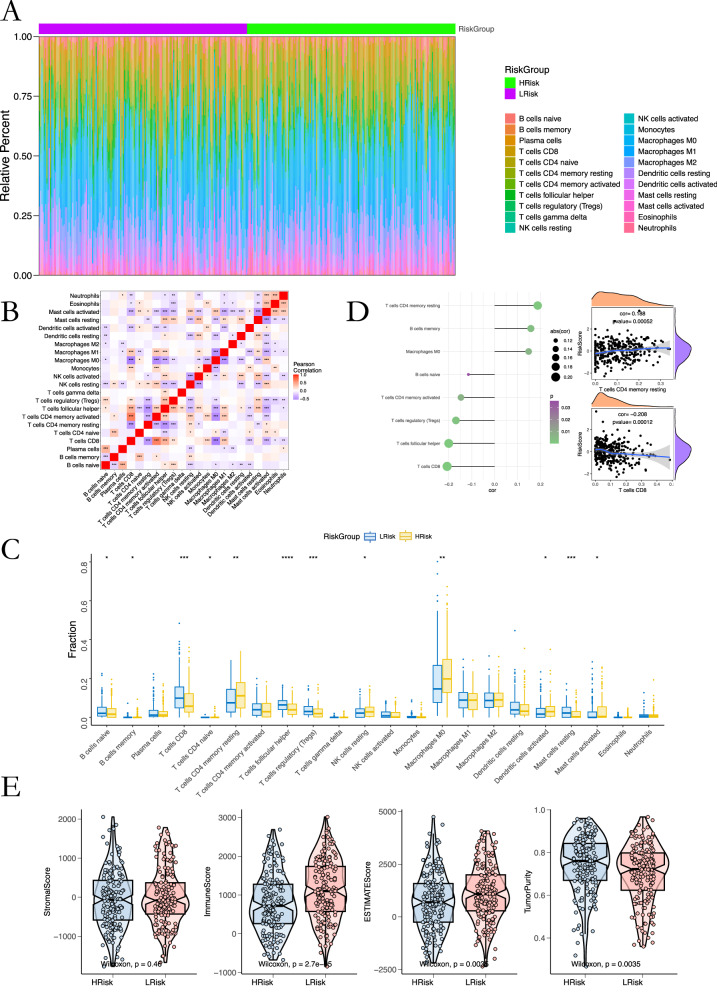
Immune cell infiltration and risk associations. **A** Distribution of 22 immune cell subsets across risk groups. **B** Correlation network among immune populations. **C** Scatter plots linking immune infiltration to risk score. **D** Differential infiltration between groups (*p* < 0.05–0.001). **E** ESTIMATE analysis showing immune/stromal component differences.

Immune deconvolution demonstrated significantly lower infiltration of CD8⁺ T cells, follicular helper T cells, and Tregs in the high-risk group, while M0 macrophages were elevated (Fig. 6A, B, D). Risk scores were inversely correlated with CD8⁺ T-cell abundance and positively correlated with M0 macrophages and resting memory B cells (Fig. 6C). ESTIMATE analysis revealed markedly reduced immune scores in the high-risk group, indicating lower immune cell infiltration and higher tumor purity (Fig. 6E). Exploratory drug-sensitivity modeling using GDSC data suggested potential differences in response to agents such as Imatinib, Bexarotene, Docetaxel, Erlotinib, and Lapatinib (Fig. A4). However, these findings are preliminary and require validation in cell-line or organoid models before clinical application. Mutation profiling identified TP53 as the most frequently mutated gene, with a higher mutation rate in high-risk tumors (82% vs. 67%), along with increased MUC16 and COL11A1 mutations, whereas NOTCH1 mutations were more common in low-risk tumors (Fig. A4).

### Immune gene expression and T-Cell dysfunction

**Fig. 7:**
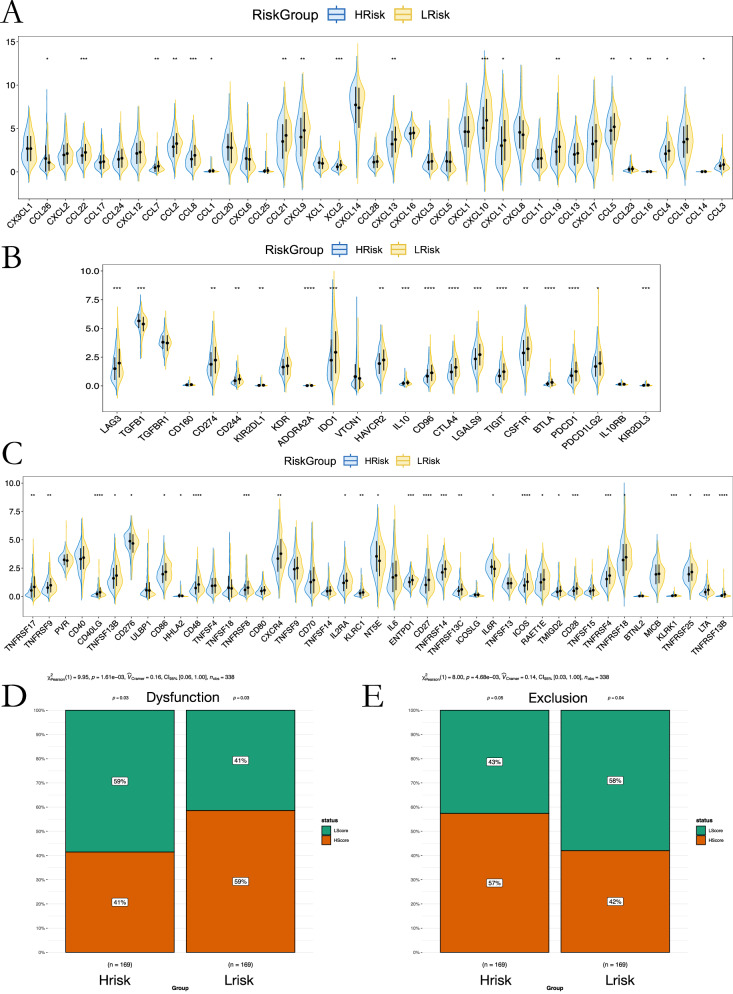
Immune factor and T-cell dysfunction patterns. **A**–**C** Chemokine, inhibitor, and stimulator expression differences between risk groups. **D**, **E** T-cell dysfunction and exclusion scores.

Immune-related gene analysis revealed downregulation of key chemokines (CCL22, CCL8, CCL21, CXCL9, CXCL10) in the high-risk group, alongside upregulation of CXCL8, a marker of poor prognosis (Fig. 7A). Immune checkpoint analysis revealed distinct expression patterns between risk groups: PD-1 (PDCD1) and CTLA-4 were upregulated in the low-risk (immune-active) group, whereas LAG3 and TIM3 were decreased in the high-risk (immune-suppressed) group. Meanwhile, CD276 and TGF-β1 were elevated in high-risk tumors, consistent with an immunosuppressive phenotype (Fig. 7B, C). These findings clarify the contrasting immune activation versus exhaustion states across the two groups. T-cell exclusion scores indicated reduced CD8⁺ T-cell infiltration potential in the high-risk group (Fig. 7D, E).

### Microbiota–immune landscape interactions

**Fig. 8:**
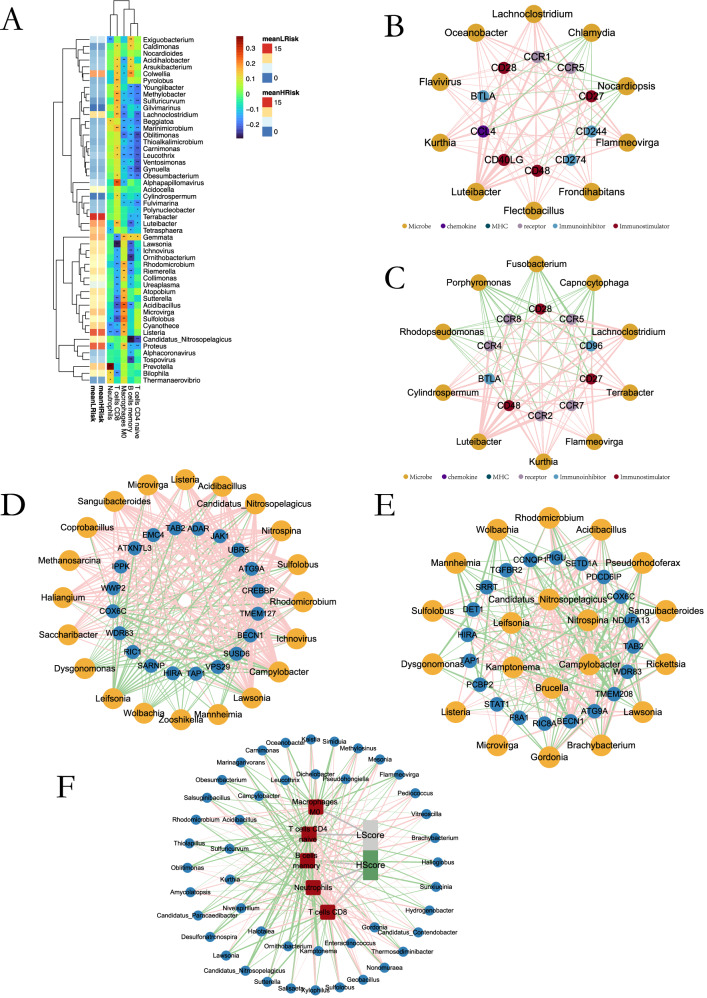
Immunomodulator–microbiome interactions. **A** Microbiome composition and immune cell infiltration correlation heatmap. **B** Immunomodulator gene-microbial abundance associations in low-risk group. **C** Immunomodulator gene-microbial abundance associations in high-risk group. **D** CTL evasion gene-microbial abundance correlations in low-risk group. **E** CTL evasion gene-microbial abundance correlations in high-risk group. **F** Intratumoral microbiome and TIDE response correlation analysis. Visualization specifications: Pink lines indicate positive correlations. Green lines indicate negative correlations. Line thickness represents correlation strength.

Microbial abundance was significantly correlated with immune infiltration patterns. Positive correlations were observed between specific taxa (e.g., *Youngiibacter*, *Methylobacter*) and M0 macrophages and naïve CD4⁺ T cells, while CD8⁺ T cells showed negative associations with multiple genera, including *Acidihalobacter* and *Colwellia* (Fig. 8A). In the low-risk group, beneficial immune factors such as CD40LG and CD48 correlated positively with *Luteibacter* and *Flectobacillus* (Fig. 8B). Periodontal pathogens, including *Porphyromonas* and *Fusobacterium*, negatively correlated with immune receptors CCR5, CCR4, and CCR8, which were downregulated in the high-risk group (Fig. 8C). Cytotoxic T-lymphocyte gene signatures were positively correlated with microbiota in low-risk tumors but negatively correlated in high-risk tumors (Fig. 8D, E). Immunotherapy response analysis revealed that high CD8⁺ T-cell infiltration clusters were associated with microbiota-driven immune activation, whereas low-CD8⁺ clusters showed microbial correlations with immune suppression, mirroring the high-risk phenotype (Fig. 8F).

## Discussion

The contribution of microorganisms to tumorigenesis has gained increasing attention in recent years, with intratumoral bacteria reported in malignancies such as lung, pancreatic, and breast cancers [16–18]. However, their impact on tumor progression, patient prognosis, and the immune microenvironment remains insufficiently understood. In this study, we identified a robust association between intratumoral microbiota and clinical outcomes in oral squamous cell carcinoma (OSCC). By constructing a predictive model based on microbial abundance, we demonstrated that specific bacterial taxa serve as independent prognostic indicators and correlate with tumor stage, lymph node involvement, and overall survival. These findings provide new evidence that intratumoral bacteria may shape OSCC biology and offer potential targets for therapeutic intervention.

The tumor–immune–microbe ecosystem (TIME) is increasingly recognized as a critical determinant of cancer progression [19]. The oral cavity, the second largest microbial reservoir after the gut, harbors over 700 bacterial species [20]. Our model revealed enrichment of *Firmicutes* and *Proteobacteria* in OSCC tissues, consistent with prior reports linking these phyla to tumor metabolic reprogramming and microenvironmental modulation [21]. We also identified strong associations between high-risk microbial profiles and genera such as *Porphyromonas* and *Fusobacterium*—taxa previously implicated in inflammation, immune evasion, and metastatic potential [22, 23]. Importantly, these genera demonstrated negative correlations with key costimulatory immune pathways (CD28, CD27, CD96, CD48) that regulate cytotoxic CD8⁺ T cells and NK cells, suggesting a potential mechanism by which intratumoral microbiota suppress antitumor immunity. This aligns with experimental evidence showing that *Porphyromonas* promotes regulatory T-cell expansion via B7-H1 induction and enhances tumor growth through the miR-21/PDCD4/AP-1 axis [24, 25], while *F. nucleatum* accelerates OSCC progression by inducing DNA damage via the Ku70/p53 pathway [26].

Our updated immune checkpoint analysis further clarified that PD-1 and CTLA-4 were more highly expressed in the low-risk (immune-active) group, whereas CD276 and TGF-β1 upregulation characterized the high-risk group, reinforcing a profile of impaired cytotoxic immunity and T-cell exclusion in poorer-outcome patients.

Our immune landscape analysis further underscores the interplay between microbial risk signatures and the tumor microenvironment. High-risk tumors exhibited markedly reduced CD8⁺ T-cell infiltration and increased M0 macrophages, alongside elevated T-cell exclusion scores and reduced immune scores. These features are indicative of an immunosuppressive milieu and align with poor patient survival. Although the M2/M1 macrophage ratio is often considered prognostic in solid tumors, recent studies have highlighted that high infiltration of M0 macrophages also correlates with advanced disease and unfavorable outcomes [27, 28], consistent with our findings.

From a clinical perspective, these results suggest that intratumoral microbial markers may complement current prognostic indices and guide treatment selection, particularly for immunotherapy-eligible patients. Salivary or tissue-based microbial detection using qPCR or metagenomic panels could provide minimally invasive monitoring tools for OSCC.

Drug-sensitivity predictions derived from GDSC modeling should be interpreted as exploratory. Future functional experiments in OSCC cell lines or patient-derived organoids are required to evaluate whether microbiota-associated signatures can predict therapeutic responses.

Despite the insights provided, several limitations must be acknowledged. The TCGA dataset lacks information on dietary habits, antibiotic exposure, systemic comorbidities, and ethnic variation, all of which can influence the oral microbiome. Additionally, while our model establishes correlation, causal mechanisms cannot be established from the current analysis. Validation studies incorporating 16S rRNA sequencing, qPCR quantification, multiplex immunofluorescence, and in situ hybridization to localize bacteria within tumor compartments are needed to confirm microbial–immune interactions [29]. Future studies across larger, geographically and behaviorally diverse OSCC cohorts will also determine whether specific bacterial signatures are linked to lifestyle factors such as tobacco, alcohol, or betel nut exposure.

## Conclusion

This study underscores the critical role of intratumoral microbiota in shaping the progression and prognosis of OSCC. Through comprehensive bioinformatic analysis, we developed and validated a robust microbial-based predictive model that enables effective risk stratification of OSCC patients. The microbial risk score demonstrated strong associations with immune cell infiltration patterns and immune evasion mechanisms, revealing a potential microbiota–immune axis in tumor biology. Integrating microbiome-based biomarkers with established clinicopathological features may enhance precision prognostication and guide personalized immunotherapeutic strategies in OSCC. Targeting microbiota-mediated immune modulation may offer novel avenues for precision medicine and improved clinical outcomes in OSCC.

## Supplementary information

Supplementary figures

## Data Availability

All data analyzed in this study are publicly available from the TCGA database.
