# Supplementary material for: Intratumoral microbiota drive immune evasion and disease progression in oral squamous cell carcinoma
Source: BDJ Open. 2025 Dec 1;11:89. doi: 10.1038/s41405-025-00385-x (PMC12669796; doi:10.1038/s41405-025-00385-x)
Supplement: Supplementary file 1 — Supplementary figures [file 41405_2025_385_MOESM1_ESM.docx]

Appendix A

**Figure A1. Taxonomic Composition and Diversity.** (A–C) Top-10 abundant phyla, classes, and genera. (D) Nine differentially abundant genera (p < 0.05, Wilcoxon). (E) Alpha-diversity indices comparing OSCC vs controls (p < 0.05).

**Figure A2.** Infiltration patterns of immune factors and their association with risk scores. Quantification of (A) MHC and (B) immune receptors, comparing their infiltration levels between the high-risk and low-risk groups. Significant differences are marked with asterisks (******P* < 0.05, ** *P* < 0.01, and *** *P* < 0.001**).**

**Figure A3.** Correlation between infiltrating immune cells and TIDE response. Differences in the infiltration levels of (A) M0 macrophages, (B) neutrophils, and (C) CD8^+^ T cells between the high-dysfunction score (Hscore)and low-dysfunction score (Lscore) groups. The *p*-value is shown at the top of the image.

**Figure A4. Chemosensitivity and mutation profiles.** (A) Drug-response (IC₅₀) differences for chemotherapeutic agents across risk groups. (B) Mutational landscape highlighting differential gene mutation frequencies by risk stratification.
